# Characterization of RBD-specific cross-neutralizing antibodies responses against SARS-CoV-2 variants from COVID-19 convalescents

**DOI:** 10.3389/fimmu.2023.1160283

**Published:** 2023-05-10

**Authors:** Zheng Wang, Dan Li, Yulu Chen, Yeping Sun, Changzhong Jin, Caiqin Hu, Yi Feng, Junwei Su, Li Ren, Yanling Hao, Shuo Wang, Meiling Zhu, Ying Liu, Jianxun Qi, Biao Zhu, Yiming Shao

**Affiliations:** ^1^ State Key Laboratory of Infectious Disease Prevention and Control, Division of Research of Virology and Immunology, National Center for AIDS/STD Control and Prevention, Chinese Center for Disease Control and Prevention, Beijing, China; ^2^ CAS Key Laboratory of Pathogen Microbiology and Immunology, Institute of Microbiology, Chinese Academy of Sciences, Beijing, China; ^3^ State Key Laboratory for Diagnosis and Treatment of Infectious Diseases, National Clinical Research Center for Infectious Diseases, Collaborative Innovation Center for Diagnosis and Treatment of Infectious Diseases, The First Affiliated Hospital, School of Medicine, Zhejiang University, Hangzhou, China

**Keywords:** SARS-CoV-2, receptor binding domain, neutralizing antibody, antibody-antigen complex, antibodyome

## Abstract

**Introduction:**

The novel severe acute respiratory syndrome coronavirus 2 (SARS-CoV-2) pandemic has been posing a severe threat to global public health. Although broadly neutralizing antibodies have been used to prevent or treat corona virus disease 2019 (COVID-19), new emerging variants have been proven resistant to these antibodies.

**Methods:**

In this study, we isolated receptor binding domain (RBD)-specific memory B cells using single-cell sorting method from two COVID-19 convalescents and expressed the antibody to test their neutralizing activity against diverse SARS-CoV-2 variants. Then, we resolved antibody-RBD complex structures of potent RBD-specific neutralizing antibodies by X-ray diffraction method. Finally, we analyzed the whole antibody repertoires of the two donors and studied the evolutionary pathway of potent neutralizing antibodies.

**Results and discussion:**

We identified three potent RBD-specific neutralizing antibodies (1D7, 3G10 and 3C11) from two COVID-19 convalescents that neutralized authentic SARS-CoV-2 WH-1 and Delta variant, and one of them, 1D7, presented broadly neutralizing activity against WH-1, Beta, Gamma, Delta and Omicron authentic viruses. The resolved antibody-RBD complex structures of two antibodies, 3G10 and 3C11, indicate that both of them interact with the external subdomain of the RBD and that they belong to the RBD-1 and RBD-4 communities, respectively. From the antibody repertoire analysis, we found that the CDR3 frequencies of the light chain, which shared high degrees of amino acid identity with these three antibodies, were higher than those of the heavy chain. This research will contribute to the development of RBD-specific antibody-based drugs and immunogens against multiple variants.

## 1 Introduction

Since late 2019, severe acute respiratory syndrome coronavirus 2 (SARS-CoV-2) has rapidly spread across the world and infected over 600 million individuals (https://COVID-19.who.int/) and caused over 6.6 million deaths (https://coronavirus.jhu.edu/map.html). Even though more than 11 billion vaccine doses have been administrated around the world, it is predictable that infection will continue to spread for a quite long time and the crisis is far from under control. Above all, SARS-CoV-2 has developed into multiple variants, including Alpha, Beta, Gamma, Delta, Lambda and Omicron (1). Compared to the WH-1 isolate, the Alpha, Delta and Omicron variants propagated faster (2, 3), and the Beta, Gamma and Omicron variants showed stronger neutralization resistance to neutralizing antibodies and vaccine-immunized sera (4–6). As a result, developing novel drugs, vaccines or neutralizing antibodies to prevent or treat COVID-19 is vital to completely contain the emergence and prevalence of SARS-CoV-2 variants.

SARS-CoV-2 is a positive strand RNA virus which belongs to the coronavirus β family (7). It encodes four structural proteins: Spike (S), Envelope (E), membrane (M), and nucleocapsid (N), as well as 16 non-structural proteins and five to eight auxiliary proteins (8). The S protein on the virus surface binds to the Angiotensin Converting Enzyme II (ACE2) on the host cells to enter the cell. S protein is subdivided into two functional units, S1 and S2 protein subunits. S1 can be divided into NTD (N-terminal domain) and RBD (receptor binding site). RBD region is about 240 amino acids long, which mainly binds to the host cell receptor, and S2 is in charge of fusing envelope and cell membrane (9). Most neutralizing antibodies target the RBD region of SARS-CoV-2 spike (S) trimer, which consists of three copies of S1 and three copies of S2. At the early stage of the SARS-CoV-2 pandemic, several neutralizing antibody candidates targeting the SARS-CoV-2 RBD were under clinical trials or approved for emergency use, including LYCoV555, REGN-COV2, TY027, CT-P59, JS016, BRII-196, BRII-198 and SCTA01 (10, 11). Although neutralizing antibodies can down-regulate the viral load, alleviate the clinical symposium and reduce the risk of disease progression in patients with mild-to-moderate COVID-19 (12, 13); however, the circulating variant, Omicron, escaped the neutralization from over 85% of tested neutralizing antibodies in a pseudovirus-based assay (14), indicating an urgent need to develop novel broadly neutralizing antibodies against emerging variants.

Currently, such neutralizing antibody isolations and identifications are mainly through antigen probes-specific sorting using flow cytometry (Fluorescence Activated Cell Sorting, FACS) or single-cell sequencing. Indeed, a few neutralizing antibodies may be missed by these methods, and it is possible to identify substantially more from the donor’s PBMCs by other methods such as next-generation sequencing technologies (15–17) or the proteomics approach (18). Antibodyomics method has been used to isolate HIV-1 broadly neutralizing antibodies, e.g., CD4bs-directed neutralizing antibodies and MPER-specific neutralizing antibodies successfully (19, 20). It is capable of identifying thousands of somatic variants of the lineage of neutralizing antibodies. Furthermore, unbiased antibody repertoire sequencing combined with established phylogenetic tools may reveal a general B cell maturation process of neutralizing antibodies against HIV or Zika (19, 21), which is helpful to provide clues to vaccine designing. Thus, it is necessary to perform antibody repertoire analysis and study the evolutionary pathway of isolated neutralizing antibodies in some COVID-19 convalescents.

In this study, we recovered three potent neutralizing antibodies by RBD-specific single B cell sorting strategy from two COVID-19 convalescents. Then, we resolved two antigen-antibody complex structures. At last, we analyzed the antibody repertoire of two donors and study the evolutionary pathway of these neutralizing antibodies. These researches will aid in the development of antibody-based drugs and immunogens that elicit RBD-specific antibodies.

## 2 Materials and methods

### 2.1 Convalescent patients and blood samples

Two COVID-19 convalescents from the first affiliated hospital, Zhejiang university school of medicine were enrolled in this study. Both convalescents were confirmed COVID-19 according to “Diagnosis and treatment of novel coronavirus pneumonia” in Jan, 2020 and hospitalized for 2-3 weeks. The discharge criteria were listed as follows. First, the body temperature turned to normal for more than three days. Second, respiratory symptoms disappeared. Third, two SARS-CoV-2-specific RT-PCR assays for consecutively throat swabs with a one-day interval turned negative. Then, five mL of whole blood were sampled in the third month following the time-point visit. Peripheral blood mononuclear cells (PBMCs) were isolated using the Ficoll-Hypaque gradient medium according to the manufacture’s instruction. PBMCs were stored in liquid nitrogen until single memory B-cell sorting. This study was approved by the Ethics Committee of the first affiliated hospital, Zhejiang University School of Medicine, China (approval number:2020-IIT-433) according to the declaration of Helsinki. Written informed consents were obtained from study participants for research use of their blood samples

### 2.2 Construction of recombinant RBD protein probe

The expression vectors for SARS-CoV-2 RBD (GenBank No: MN908947) which carries the Avi-tag sequence for biotinylation at the 3’ end of the gene were constructed using the vector pDRVI1.0 (22, 23). After expression and purification, the proteins were biotinylated by utilizing a biotin ligase Bir A500 kit (Avidity, USA) to conjugate with the streptavidin-fluorochrome reagents as previously described (24). Further, Streptavidin-phycoerythrin (SA-PE) (E4011, Sigma, USA) was mixed with biotinylated RBD as previously reported to do single B cell sorting (25).

### 2.3 Measurement of anti-RBD antibody response using ELISA

Briefly, 2μg/ml SARS-CoV-2 RBD was coated onto polystyrene 96-well microplates and incubated at 4°C overnight. The plates were washed with PBST (PBS containing 0.2% Tween 20) and were blocked using 2% BSA for two hours at room temperature. After washing with PBST, 100 μL of serially diluted plasma samples or isolated monoclonal antibodies were added to each well and incubated at 37 °C for one hour. Each well was then incubated with 100 μL of diluted secondary anti-human IgG labeled with HRP (Cat. BF03027, Biodragon, China) for one hour after washing. TMB substrate (100 μL/well) was subsequently added and incubated for five minutes after PBST washing and the reaction was stopped by adding 50 μL/well of 2 M H_2_SO_4_. Finally, optical density (OD) was measured by a spectrophotometer at 450 nm and 630 nm.

### 2.4 Evaluation of neutralizing activity of plasma or antibody against SARS-CoV-2 pseudoviruses

Pseudovirus neutralization assay was conducted as described previously (26). Briefly, SARS-CoV-2 pseudovirus was generated by transfecting SARS-CoV-2 S protein expression plasmid (pcDNA3.1. S2) into 293T cells. At the same time, 293T cells were infected with VSV G pseudotyped virus, in which the G gene was replaced by a luciferase reporter gene. Viral supernatants were collected 48 hours later and were titered on Vero cells based on a chemiluminescence detection method (Cat. 6066769, Promega, USA) to get a 50% tissue culture infectious dose (TCID_50_) value according to the Reed-Muench method. For neutralization assays, 50 μL of pseudovirus (100 TCID_50_) was incubated with 50 μL of serial dilutions of samples at 37 °C for one hour. Then 100 μL of Vero cells (approximately 10^4^ cells per well) were added in duplicate to the virus–antibody mixture. The cell control with only cells as well as the virus control with virus and cells are also set up in each plate. Half-maximal inhibitory concentrations (IC_50_) of the evaluated monoclonal antibodies were determined by measuring luciferase activity 24 hours post-infection.

### 2.5 Isolation of RBD-specific memory B cells by single-cell sorting

Briefly, cryopreserved PBMC were thawed in pre-warmed RPMI 1640 (Cat. 12019003, Corning, USA) with 10% fetal bovine serum and 50 IU/ml benzonase (Sigma, USA). Thereafter, PBMCs were washed, surface stained with antibody cocktail containing following antibodies: anti-CD3-Alexflour 700, anti-CD8-Pacific Blue, anti-CD14-Pacific Blue, anti-CD19-PECy7, anti-CD27-APCCy7, anti-IgG-FITC, anti-IgM-PECy5 (above antibodies are all from BD Biosciences), anti-CD20-ECD (Beckman Coulter) and anti-RBD-PE in a total volume of 50 μL on ice in dark for one hour, followed by Live/Dead staining with a LIVE/DEAD Fixable Aqua Dead Cell Stain Kit (Cat. L34957, Invitrogen, USA). The cells were washed and suspended in cold PBS with 2mM EDTA. The stained PBMC were filtered by 70-μm cell mesh (Cat. 352235, Corning, USA) and sorted using a five-laser FACS Aria cell sorter III driven by FACS Diva software. Single cells with the phenotype of CD3-, CD8-, DAPI-, CD14- CD19+, CD20+, CD27+, IgM-, IgG+, RBD+ were defined as SARS-CoV-2 RBD specific memory B cells. Single cells were sorted into 96-well PCR plates containing 20 μL of cell lysis buffer per well under yield mode, and PCR plates were quickly frozen on dry ice and stored at -80˚C overnight. Then RT-PCR were performed to amplify the variable regions of the heavy chain (V_H_) and light Chain (V_L_) as previously reported (27).

### 2.6 Antibody expression and purification

V_H_ and V_L_ were cloned into the CMV/R expression vector containing the constant regions of IgG1 heavy chain or Light chain (25). The paired IgG Heavy chain plasmids and light chain plasmids were co-transfected into 293F cells, the cells were incubated at 37 °C in a humidified 8% CO_2_ environment for five days, then the supernatant was collected and antibodies were purified using a recombinant protein-A column (Cat.20415057, Senhui Microsphere, China). The antibody concentration was determined by Nanodrop 2000 ultramicro spectrophotometer (Thermofisher, USA) and stored at 4 °C for detection.

### 2.7 Measurement of antibody binding kinetics

Antibody-antigen kinetics (K_D_) was measured by an Octet^®^ Red 96 machine (ForteBio, USA). Briefly, biotylated RBD of SARS CoV-2 was diluted to a concentration of 5 μg/mL with PBST (PBS containing 0.02% Tween 20 and 0.1% BSA) and then immobilized onto streptavidin biosensors (Cat.18-5019, Fortebio, Germany) for 60 seconds. After a 60 seconds wash step with PBST, biosensor tips were immersed into the wells containing serially diluted antibodies (500 nM, 250 nM, 125 nM, 62.5 nM, 31.25 nM, 15.625 nM and 7.8125 nM) and associated for 120 seconds, followed by a 300 seconds dissociation step. The K_D_ values were calculated using a 1:1 binding model in Data Analysis Software 9.0.

### 2.8 Neutralizing activity of mAbs against live SARS-CoV-2

SARS-CoV-2 obtained from a sputum sample was amplified in Vero-E6 to make working stocks of the virus. To analyze the mAbs’ neutralizing activity, two-fold serial dilutions of mAbs were added to the same volume of 100 TCID_50_ of SARS-CoV-2 in quadruplicate and incubated for 1 hour at 37˚C. The mixture was added to a monolayer of Vero-E6 cells in a 96-well plate and incubated at 37˚C. Cytopathic effects (CPE) were observed and recorded on day 5. The antibody concentration that cytopathic effect presented in half wells was defined as IC_50_.

### 2.9 Unbiased antibodyomics analysis

An RNeasy mini kit (Cat.74104, Qiagen, Germany) was used to extract total RNA from donor CZ and donor WJQ PBMCs. Then, using a SMARTer Race 5’/3’ Kit (Cat.634858, Takara, Japan), unbiased antibody library containing V_H_, variable regions of kappa chain (Vκ) and variable regions of lambda chain (V_λ_) were prepared as previously described, respectively (21). The reverse primers sequences are as follows: V_H_:5’-GGGGAAGACCGATGGGCCCTTGGT -3’, Vκ: 5’-CAGCAGGCACACAACAGAGGCAGTTCC -3’ and V_λ_: 5’- CACCAGTGTGGCCTTGTTGGCTTG -3’. After Illumina Miseq PE300 sequencing, data processing and error correction, the sequencing data were initially evaluated using IMGT/HighV-QUEST online tool (http://imgt.org/HighV-QUEST/index.action), and further analyzed with phylogenetic tree and related R package.

### 2.10 Crystallization, data collection and structure determination

The crystals of 3G10/RBD were harvested in 0.2 M L-Proline, 0.1 M HEPES pH 7.5, 10% w/v Polyethylene glycol 3350, while the crystals of 3C11/RBD were harvested in 0.1 M magnesium chloride, 0.1 M Na HEPES, pH 7.5, 10% w/v PEG 4000. To collect the diffraction data, complex crystals were briefly soaked in the corresponding reservoir solutions containing 20% (v/v) glycerol for cryo-protection and then flash-cooling in liquid nitrogen. The diffraction data were collected at the Shanghai Synchrotron Radiation Facility (SSRF) BL02U1. The datasets were processed using HKL2000 software (28). The structures of these three complexes were determined *via* the molecular replacement method using Phaser (29) with a search model previously reported (PDB: 6LZG). The atomic models were built using Coot (30) and refined with phenix.refine (29). MolProbity tool (31) was used to assess the stereochemical qualities of the final models. Finally, all structures were generated using Pymol software (https://pymol.org/2/).

## 3 Results

### 3.1 Characterization of plasma of COVID-19 convalescents

The whole blood of two COVID-19 convalescents was collected, who recovered from a common type symptom and a severe type symptom, respectively (**Table 1**). The RBD-specific response of plasma was measured by ELISA, and the end-titers against RBD were determined as 1:100 and 1:300, respectively (**Table 1**, **Figure S1A**). To confirm the presence of potent neutralizing antibody in the plasma, SARS-CoV-2 pseudovirus of Wuhan stain (WH-1) was used to evaluate the NT_50_ of plasma. The plasma neutralized the pseudovirus with NT_50_ value of 356.6 and 317.7 (reciprocal dilution), respectively (**Table 1**, **Figure S1B**). The results indicate that both the two donors possess the RBD-specific antibodies and neutralizing antibodies against SARS-CoV-2.

**Table 1:** Background information of two donors.

Patient No	Age	Clinical symptom	Gender	Hospitalization Length (days)	RBD-specific antibody titer	Neutralizing activity (NT_50_)
CZ	34	Severe	Male	19	1:100	1:356.6
WJQ	24	common	Female	14	1:300	1:317.7

### 3.2 Isolation of RBD-specific antibodies

**Figure 1:**
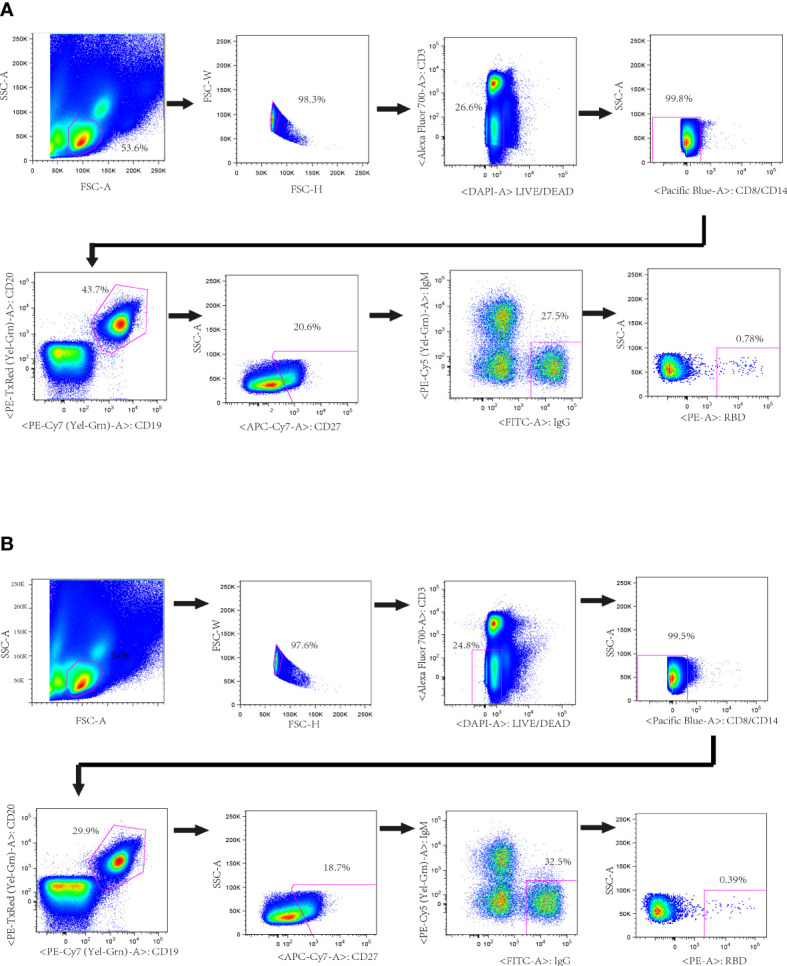
Isolation of probe-specific memory B cells from PBMCs of convalescent donors. RBD-specific B cell sorting by flow cytometry from donors’ PBMCs. About 10 million PBMCs were incubated with cocktail antibodies and RBD-PE probe. Memory B cells (CD19^+^, CD20^+^ and IgG^+^) that bound to RBD-PE probe were sorted into a 96-well plate containing lysis buffer. The percentages of IgG+ B cells that reacted with RBD are indicated. **(A)** Donor CZ **(B)** Donor WJQ.

**Figure 2:**
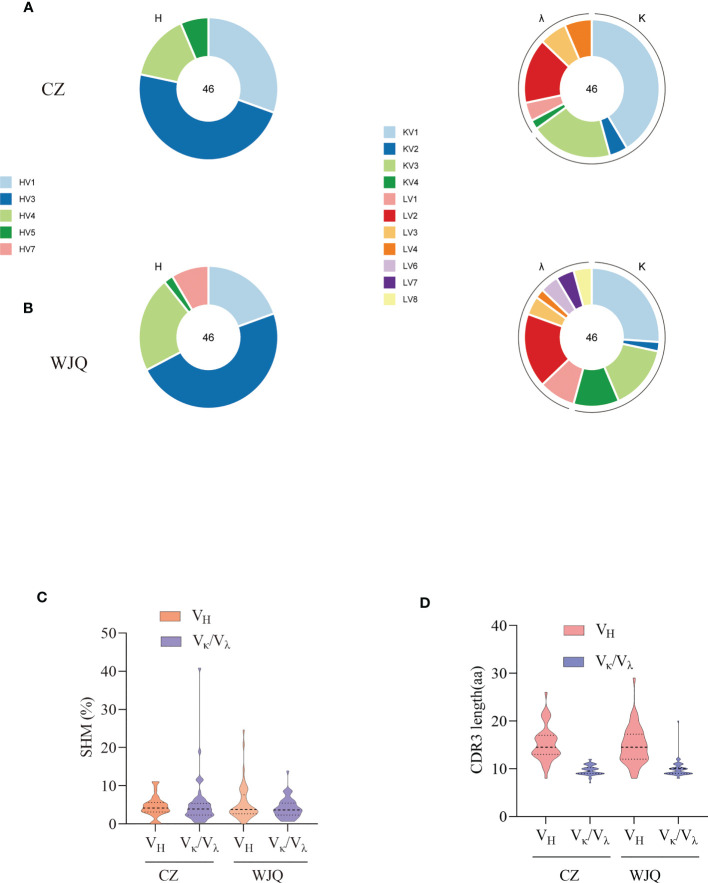
Characterization of sequence features of isolated antibodies of two convalescent donors. **(A)** Germline distribution of V_H_ and Vκ/V_λ_ of isolated antibodies of donor CZ. **(B)** Germline distribution of V_H_ and Vκ/V_λ_ of isolated antibodies of donor WJQ. **(C)** SHM rates of V_H_ and V_L_ of isolated antibodies. **(D)** CDR3 lengths of V_H_ and Vκ/V_λ_ of isolated antibodies.

To isolate the RBD-specific memory B cells, 10^7^ PBMCs of each donor were stained by cocktail antibodies plus PE-labeled RBD probe and sorted by a FACS Aria Cell Sorter III machine. One hundred and thirty-seven and two hundred and twenty-three single B cells were isolated from donor CZ and donor WJQ respectively and subjected to a V_H_/V_L_-specific PCR amplification subsequently (**Figure 1**). In detail, 46 paired V_H_ and V_L_ genes of each donor were acquired, sequenced and analyzed sequentially. As shown in **Figure 2**, V_H_ genes originate from IGHV1, IGHV3, IGHV4, IGHV5 and IGHV7 germlines, and IGHV3 presents the highest proportion—47.8% —in both donors. For V_L_ genes in donor CZ, IGKV1and IGLV2 account for 61.2% in κ chains and 48% in λ chains respectively. Similarly, these two germlines make up 48% and 38.1% in donor WJQ respectively.

Somatic hypermutation rate (SHM) and CDR3 length are critical criteria for determining antibody maturation. The average SHM rates of V_H_/V_L_ of two donors are both approximately 5%, while median CDR3 lengths of V_H_/V_L_ present 15 amino acids (aa) and 10 aa in two donors, respectively (**Figure 2**).

V_H_/V_L_ genes were digested by restriction enzymes and ligated into the IgG expression vectors. Then antibodies were produced by transfecting paired V_H_ and V_L_ expression vectors into 293F cells and were purified using protein A column. Following ELISA screening, 61 RBD-specific antibodies were identified as positive and their neutralizing activity was evaluated using a SARS-CoV-2 pseudovirus (WH-1) (**Figure S2**). Among these antibodies, 1D7 from donor CZ, and 3G10 and 3C11 from WJQ had IC50 values of less than 0.1 μg/mL, and they were considered potent neutralizing antibodies. The alignments of the V_H_ and V_L_ genes of these three antibodies and respective germline genes are shown in **Figure S3**. The V_H_ of 1D7, 3G10 and 3C11 belongs to IGHV1-69*09F, IGHV3-66*02F and IGHV3-48*03F respectively. The V_L_ of 1D7, 3G10 and 3C11 originates from IGKV3D20*01F, IGKV1-9*01F and IGKV3-11*01F, respectively. SHM rates of the V_H_ of 1D7, 3G10 and 3C11 are 4.86%, 3.86%, and 4.17%, respectively, and lengths of the HCDR3 are between 11 and 18 aa. SHM rates of the V_L_ of these three antibodies are around 2.5%. In particular, CDR3 of light chain (LCDR3) lengths of the three antibodies are all nine aa (**Figure S3**).

### 3.3 Binding activity of 3G10 and 3C11 with RBD

**Figure 3:**
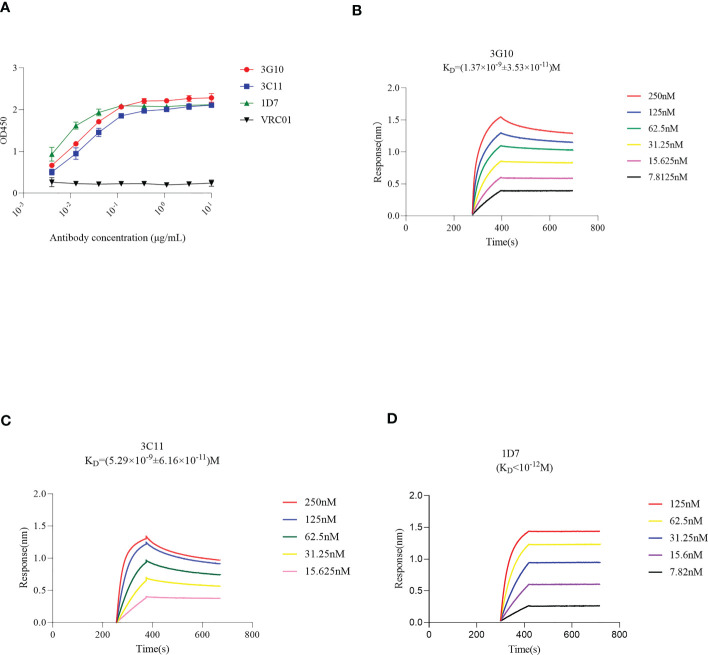
Binding activity of antibodies 3G10, 3C11 and 1D7 with RBD, using ELISA or BLI method. Briefly, biotinylated RBD was immobilized onto SA sensors followed by association of mAbs at different concentrations and dissociation subsequently. The sensorgrams show binding patterns of mAbs with RBD. K_D_ values were calculated with a 1:1 binding model using Data Analysis Software 9.0. **(A)** RBD-specific response of 3G10, 3C11 and 1D7 using ELISA test. HIV Env –specific antibody VRC01 was used as an isotype control. **(B-D)** Binding kinetics of 3G10, 3C11 and 1D7 with RBD with Octet Red 384, respectively.

ELISA was used to test the binding activity of 1D7, 3G10, and 3C11 with RBD, and the EC_50_ values were 0.004 μg/mL, 0.015 μg/mL and 0.019 μg/mL, respectively (**Figure 3A**). Furthermore, the Biolayer-interferometry method (BLI) approach was used to assess the binding kinetics of antibodies and RBD. **Figures 3B-D** show that 3G10, 3C11 and 1D7 bind RBD with K_D_ values of (1.37×10^-9^ ± 3.53×10^-11^) M, (5.29×10^-9^ ± 6.16×10^-11^) M, and less than 10^-12^ M respectively. These findings suggest that these three antibodies have high affinity with RBD.

### 3.4 Neutralizing activity of 1D7, 3G10 and 3C11 against SARS-CoV-2 pseudovirus variants

**Figure 4:**
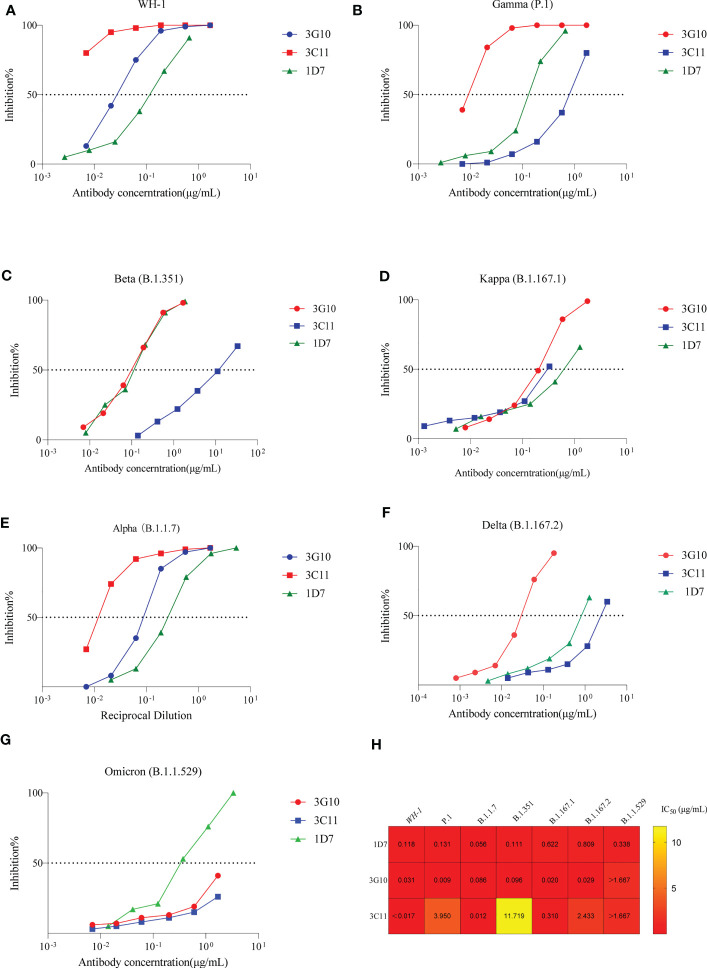
Neutralizing activity of 3G10, 3C11 and 1D7 against SARS-CoV-2 variants pseudoviruses. Antibody concentration and inhibitory effectiveness are shown on the X- and Y-axes, respectively. **(A)** SARS-CoV-2 WH-1 strain. **(B)** P.1 variant. **(C)**B.1.351 variant. **(D)** B.1.617.1 variant. **(E)** B.1.1.7 variant. **(F)** B.1.617.2 variant. **(G)** Omicron variant (B.1.1.529). **(H)** Heat map of IC50 values of antibodies against SARS-CoV-2 variants pseudoviruses.

Furthermore, neutralizing activity of antibodies against circulating virus variants, e.g., Alpha, Beta, Kappa, Delta, Gamma and Omicron was tested (**Figure 4**). 1D7 potently neutralized all variant (IC_50_ values<1μg/mL). Except for Omicron, 3G10 could efficiently neutralize variants with IC_50_ values less than 0.1 μg/mL. As to 3C11, it neutralized Alpa and Kappa variants with IC_50_ values of 0.012 μg/mL and 0.31μg/mL, respectively, but neutralized P.1 with a higher IC_50_ value of 3.95 μg/mL. 3C11, in particular, did not inhibit the Beta and Omicron variants.

Next, we evaluated the neutralizing activity of these three antibodies against Omicron variants, BA.5, BF.7 and XBB, respectively. The results were shown in **Figure S7**; none of the antibodies could neutralize any one of the three variants.

### 3.5 Neutralization activity of 1D7, 3G10 and 3C11 against authentic SARS-CoV-2 virus

**Figure 5:**
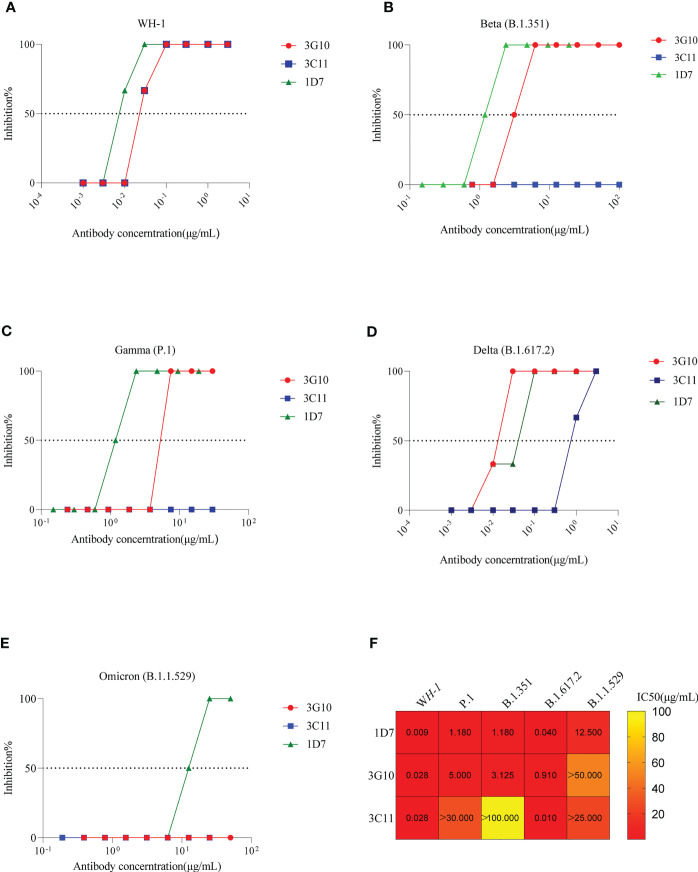
Neutralizing activity of 3G10, 3C11 and 1D7 against live SARS-CoV-2 variants. The X- and Y-axis indicate antibody concentration and inhibition efficiency respectively. **(A)**WH-1. **(B)**B.1.351. **(C)** P.1. **(D)** B.1.617.2. **(E)** B.1.1.529. **(F)** Heat map of IC50 values of antibodies against SARS-CoV-2 variants.

The neutralizing capacity of these three antibodies against authentic SRAS-CoV-2 virus (WH-1, Beta, Gamma, Delta and Omicron) was evaluated using a CPE-based method on Vero cells. As **Figure 5** shows, 1D7, 3G10 and 3C11 efficiently neutralized the WH-1 virus with IC_50_ values of 0.009 μg/mL, 0.028μg/mL and 0.028 μg/mL, respectively. However, 3C11 presented poor neutralization capacity except for WH-1 and delta variant. Antibody 3G10 neutralized tested variants except for Omicron and presented higher IC_50_ values (>3 μg/mL) against Beta and Gamma variants. For antibody 1D7, it potently neutralized all the tested viruses, but only neutralized Omicron with a IC_50_ value of 12.5 μg/mL. These results indicated that 1D7 was a broadly neutralizing antibody.

### 3.6 Crystal structures of 3G10/RBD and 3C11/RBD complexes

To probe the molecular basis of 3G10 and 3C11 binding to SARS-CoV-2 RBD, we solved the crystal structures of 3G10/RBD and 3C11/RBD complexes. The two complexes were solved at resolutions of 2.07 Å and 3.0 Å, respectively. There are two closely packed 3G10/RBD complex molecules in the asymmetric unit of the 3G10/RBD crystal structure, but only one 3C11/RBD complex molecule in the 3C11/RBD crystal asymmetric unit.

**Figure 6:**
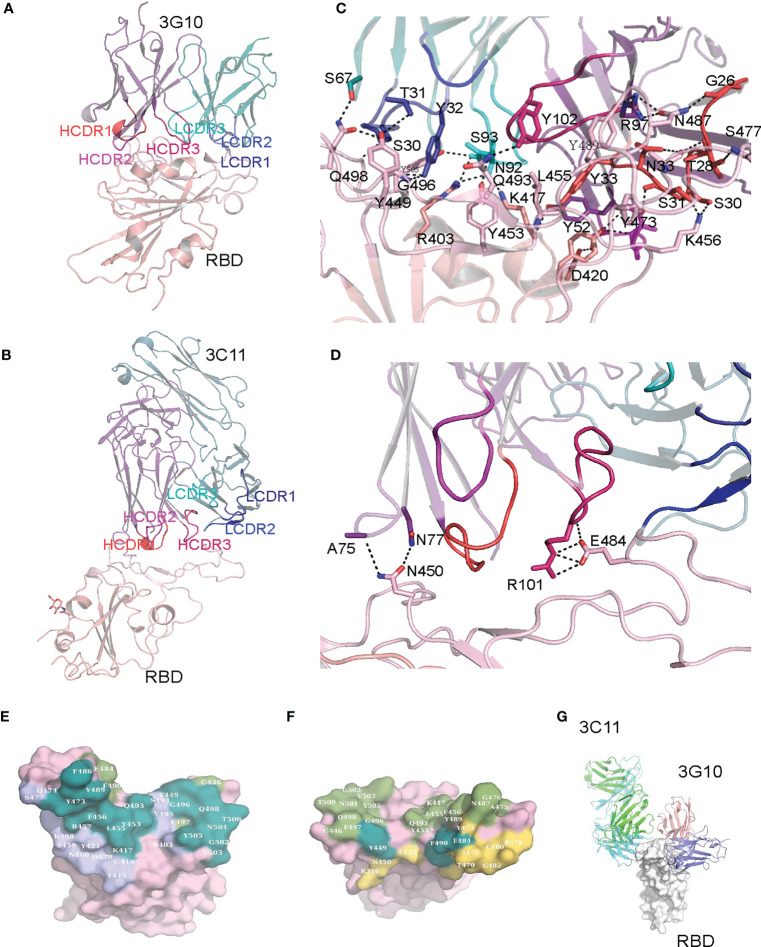
Crystal structures of 3G10/RBD and 3C11/RBD complexes. **(A)** Crystal structure of the 3G10-RBD complex. **(B)** Crystal structure of the 3C11-RBD complex. **(C)** Hydrogen bonds between the 3G10 and RBD. **(D)** Hydrogen bonds between 3C11 and RBD. **(E)** Comparison of the footprint of 3C10 and ACE2 on RBD. The RBD residues that contact both 3C10 and ACE2, those contact only ACE2, and those contact only 3C10 are colored with deep steal, smudge and slate, correspondingly. **(F)** Comparison of the footprint of 3G11 and ACE2 on RBD. The RBD residues that contact both 3G11 and ACE2, those contact only ACE2, and those contact only 3G11 are colored with deep steal, smudge and yellow orange, correspondingly. The 3G10 and ACE2 contact residues **(E)** or the 3C11 and ACE2 contact residues **(F)** are mapped on the surface representation of RBD in the RBD/ACE2 complex (PDB ID: 6lzg). **(G)** Comparison of the binding positions on 3G10 and 3C11.

Both 3G10 and 3C11 interact with the external subdomain of the RBD. In the 3G10/RBD complex, all the six CDRs of the heavy and light chains of 3G10 are involved in interactions with RBD external subdomain (**Figure 6A**). By contrast, in the 3C11/RBD complex, only CDR2 and CDR3 of the 3C11 light chain, and the CDR1 and CDR3 of the 3C11 heavy chain have interactions with RBD external subdomain (**Figure 6B**). Accordingly, 3G10 forms much more numbers of atom contacts than 3C11. In 3G10/RBD, 31 3G10 residues form 381 pairs of atom contacts with 32 RBD residues, while in 3C11/RBD, only 18 3C11 residues form 169 pairs of atom contacts with 14 RBD residues (**Table S1**, **Table S2**). Furthermore, 3G10 forms an extensive hydrogen bond network with the RBD, while only a few hydrogen bonds form between 3C11 and the RBD (**Figures 6C, D**). However, 3C11 R101 forms salt bridges with RBD E484 (**Figure 6D**). Salt bridges are the strongest non-covalent interactions between proteins, which may explain the fact that the KD between 3C11 and RBD is comparable to that between 3G10 and RBD although the total number of atom contacts between 3C11 and RBD is much less than that between 3G10 and RBD.

Many RBD residues that interact with 3G10 overlap with those which interact with the ACE2 receptor in the RBD/ACE2 complex structure (PDBID: 6LZG). These RBD residues include K417, Y449, Y453, L455, F456, Y473, A475, G476, F486, N487, Y489, Q493, G496, Q498, T500, N501, G502 and Y505 (**Figure 6E**). This means that 3G10 almost completely blocks the binding site of ACE2 on the RBD and thus repels RBD binding to ACE2. In contrast, only three RBD residues (Y449, E484, and F490) that interact with 3C11 also interact with ACE2 (**Figure 6F**). Even so, the binding of 3C11 to the RBD still generates steric hindrance which prevents the RBD from approaching ACE2. Of note, 3G10 and 3C11 bind to different sites on the RBD external subdomain. They bind to the opposite sides of the RBD (**Figure 6G**). According to the classification rule of RBD-specific antibodies (32–34), 3G10 and 3C11 belong to the RBD-1 and RBD-4 communities, respectively.

### 3.7 Unbiased analysis of B cell repertoires of donors

To study the evolution routes of neutralizing antibodies of this two donors, V_H_, Vκ and V_λ_ were unbiasedly amplified with 5’RACE PCR and sequenced using Illumina Miseq V3 machine sequentially. After assembling and clearing, 248, 753 V_H_ sequences, 370, 366 Vκ sequences and 166, 695 V_λ_ sequences covering variable regions of donor CZ were acquired;248, 197 V_H_ sequences, 342, 862 Vκ sequences and 300, 178 V_λ_ sequences were acquired from donor WJQ. Their germline distributions were mapped as **Figure S4A** and **Figure S5A**. For donor CZ, IGHV4-39, IGKV3-15 and IGLV2-14 germlines present the highest percentages in the heavy chain germlines, the kappa chain germlines and the lambda chain germlines, respectively. As to donor WJQ, IGHV4-59, IGKV4-1 and IGLV2-14 germlines have the highest frequencies among the three V_H_/Vκ/V_λ_ germlines, respectively.

The SHM rates of V_H_ and Vκ/V_λ_ of donor CZ cover from 1%-17%, and donor WJQ has similar SHM rates of V_H_ and Vκ/V_λ_ to donor CZ (**Figure S4B**. **S5B**). Regarding the CDR3 length, V_H_ mainly focuses on 12-22 aa while Vκ/V_λ_ basically centralize between 10-13 aa, both in donor CZ and donor WJQ (**Figure S4C**, **S5C**).

Furthermore, we also analyzed the constituent ratio of each J region that was related to the antibody recombination. For donor CZ, IGHJ4 (54.5%), IGHJ5 (16.5%) and IGHJ6 (16.3%) are mainly involved in the heavy chain rearrangement; IGKJ1 (27.4%), IGKJ2 (29%) and IGKJ4 (24%) principally participated in the kappa chain rearrangement; IGLJ1, IGLJ2 and IGLJ3 accounted for 19%, 45.4% and 34.5% respectively in the whole IGLJ family that referred to the recombination (**Figure S6A**). Similarly, in donor WJQ, IGHJ3 (13.1%), IGHJ4(44.2%), IGHJ5(17.9%)and IGHJ6(19.3%)dominated in the heavy chain; IGKJ1, IGKJ2 and IGKJ4 occupied 30.6%, 26.1% and 23.6% respectively in the kappa chain; IGLJ1 (14.7%), IGLJ2(30.8%) and IGLJ3(53.6%) mainly participated in the lambda chain arrangement (**Figure S6B**).

### 3.8 Phylogenetic analysis of neutralizing antibodies from antibody repertoire

**Figure 7:**
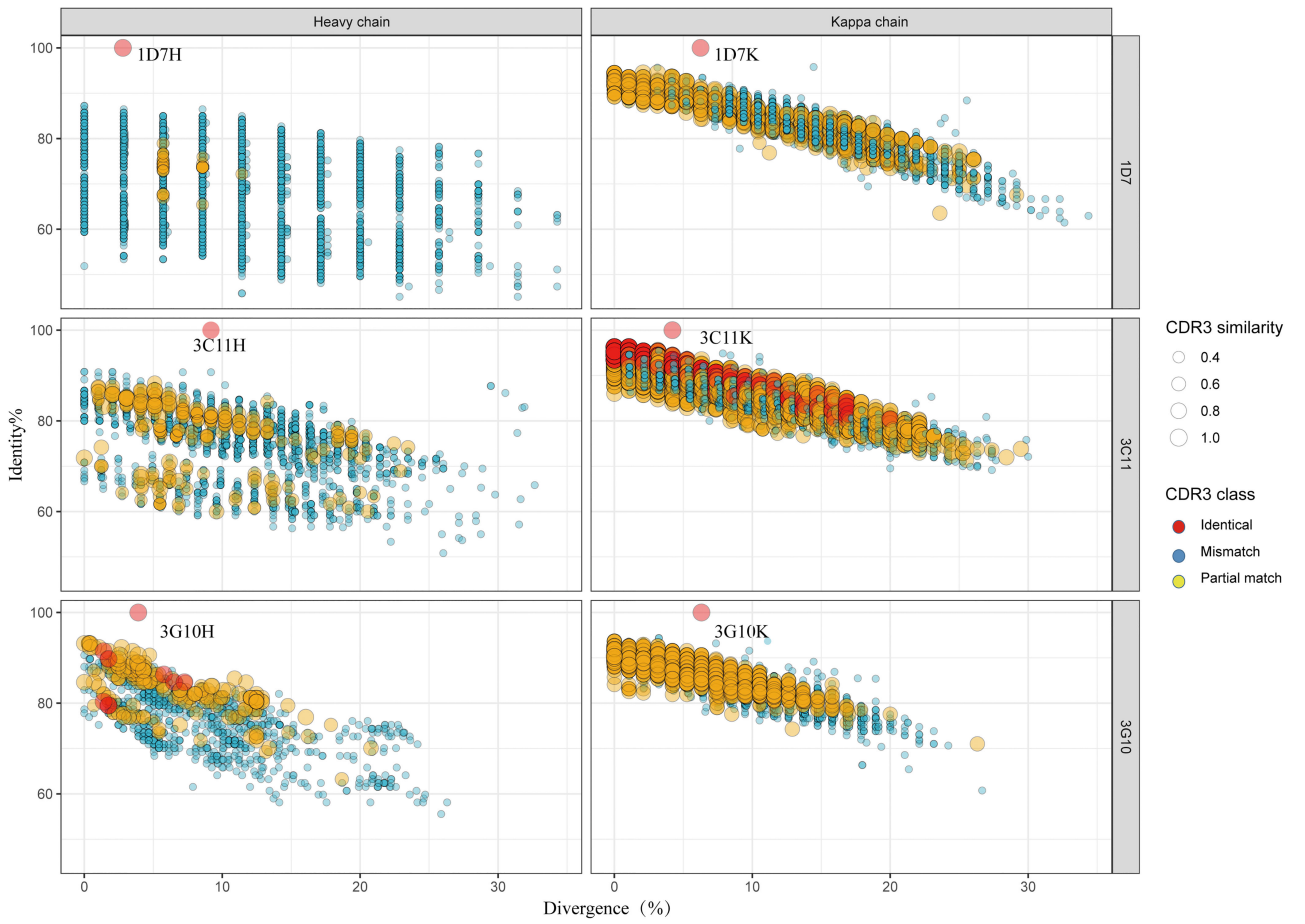
Identity-divergence analysis of the neutralizing antibodies of the unbiased heavy (H) and light (κ) chain repertoires from the two donors. The x-axis and y-axis indicate the sequence diversities to the germline and the sequence identities to the isolated antibody, respectively. The size of the circle indicates the CDR3 similarity to the target antibody. The color in the circle divides the CDR3 similarity to the target antibody into three classes: Identical (red, 100%), Mismatch (blue, ≤40%) and Partial match (yellow, 50%-90%). Analyses of antibodies 1D7, 3C11 and 3G10 were arrayed from top to bottom of the figure, separately.

The identity-divergence two-dimensional (2D) plots were used to analyze the V_H_/Vκ repertoires with respect to 1D7, 3C11 and 3G10 antibodies, respectively (**Figure 7**). Briefly, 12, 431 V_H_ sequences and 6, 442 Vκ sequences of V_H_/Vκ germlines of 1D7, 1, 614 V_H_ sequences and 10, 457 Vκ sequences of V_H_/Vκ germlines of 3G10, and 4, 607 V_H_ sequences and 30, 826 Vκ sequences of V_H_/Vκ of 3C11 germlines were identified from the antibody repertoires. The cut-off value for identifying sequences phylogenetically related to 1D7, 3C11 or 3G10 was an 85% HCDR3/LCDR3 identity to V_H_/Vκ of these three antibodies. The related V_H_ of 1D7 and 3C11 were not directly found in the repertoires for no high identity (over 85%) HCDR3 sequences were identified (**Figure 7**). On the other hand, 72 sequences were phylogenetically related to the Vκ of 1D7, and 2, 288 sequences were phylogenetically associated with the Vκ of 3C11 (**Figure 7**). Regarding antibody 3G10, seven sequences and six sequences were phylogenetically related to the V_H_ and Vκ of 3G10, respectively.

**Figure 8:**
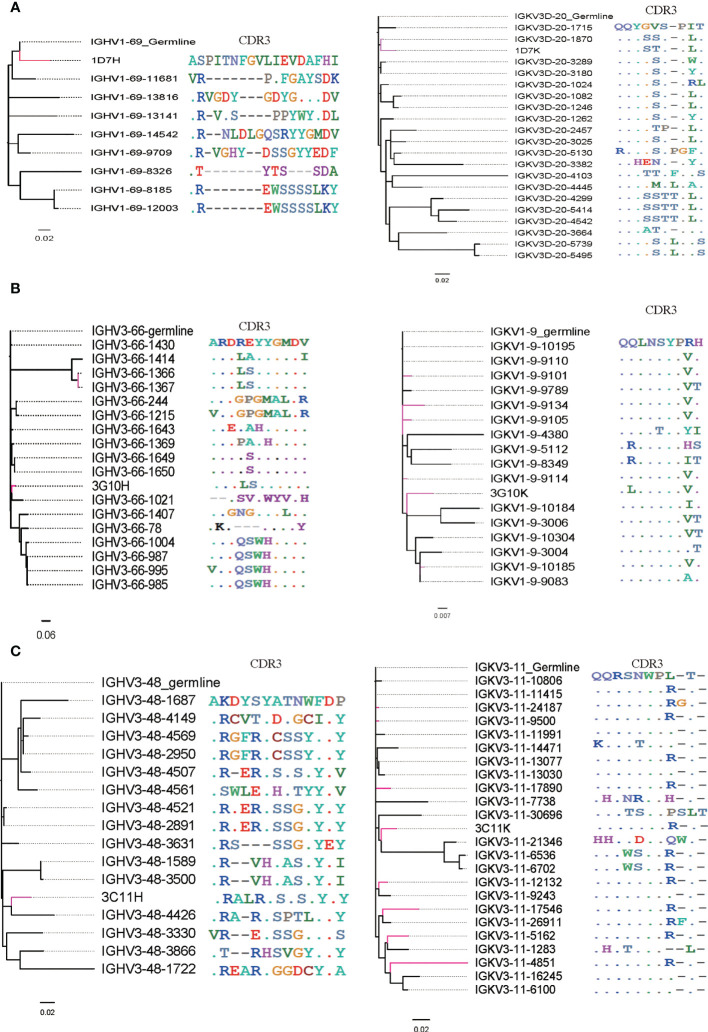
Phylogenetic analysis and CDR3 alignment between V_H_/Vκ of three neutralizing antibodies and selected V_H_/Vκ sequences from their corresponding germline allelic genes. V_H_/Vκ of antibodies 3G10, 3C11 and 1D7 and sequences with high CDR3 identity are marked red. **(A)** V_H_/Vκ of 1D7, **(B)** V_H_/Vκ of 3G10 and **(C)** V_H_/Vκ of 3C11.

To further study the evolutionary route of 3G10, 3C11 and 1D7, we chose representative sequences based on the CDR3 identity from repertoires and analyzed them by phylogenetic trees (**Figure 8**). All the selected antibody variants that are related to the three antibodies have low degrees of V_H_/Vκ divergences (<5%). The CDR3 frequencies of Vκ, which share high degrees of aa identity with these three antibodies, are higher than those of V_H_. It implies that V_H_ experiences a greater selective pressure than Vκ during affinity maturation, which is consistent with the notion that V_H_ play a more important role in the RBD binding than Vκ, as suggested by the structures of RBD-antibody complexes.

## 4 Discussion

In this research, we isolated three neutralizing antibodies—1D7, 3G10 and 3C11— from two convalescents who recovered from the common or severe clinical symposium. These three antibodies could bind RBD with high avidity and effectively neutralized the SARS-CoV-2 live virus (WH-1 and Delta strains). 1D7 antibody, in particular, demonstrated neutralizing activity against four circulating variants, the South Africa variant, Brazil variant, Delta variant and Omicron variant, and could be used to treat or prevent SARS-CoV-2 infection.

At the early stage of the SARS-CoV-2 pandemic, several neutralizing antibodies targeting RBD or NTD were quickly isolated and were found to potently inhibit WH-1 virus strain *in vivo* or *in vitro*. Some of them have been accepted into the clinical trial stage (13, 35, 36). Following that, several SARS-CoV-2 variants emerged, including the Beta variant, the Gamma variant, the Kappa variant, the Delta variant, and the Omicron variant, raising global public health concerns. Delta and Omicron variants are currently the most common circulating variants worldwide. Previous research found that these variants were more resistant to neutralizing antibodies and vaccine-immunized plasma (5, 37, 38). Omicron escaped over 85 percent of tested neutralizing antibodies, including LY-CoV016, LY-CoV555, REGN10933, REGN10987, AZD1061, AZD8895, and BRII-196 (14). In our study, we discovered that 3G10, 3C11, and 1D7 antibodies effectively neutralized WH-1, B.1.1.7, B.1.167.1, and B.1.167.2 pseudoviruses, as well as WH-1 and B.1.167.2 live viruses. The 1D7 antibody, in particular, demonstrated neutralizing activity against all of the tested viruses, including the Omicron variant, indicating that it could be used to treat or prevent SARS-CoV-2 infection. It’s worth noting that we discovered some differences in neutralization results between pseudotyped and authentic viruses. Because of the different assay systems and procedures, pseudoviruses have lower IC50 values than authentic viruses.

The crystal structures of 3G10/RBD and 3C11/RBD complexes reveal that both 3G10 and 3C11 interact with the external subdomain of the RBD, although they bind to different sites. This suggests that both of the antibodies may competitively inhibit RBD binding to the ACE receptor. The interface assay shows that 3G10 forms very extensive atom contacts with RBD, which supports the high affinity between 3G10 and RBD determined by the BLI assay. Although the total number of atom contacts between 3C11 and RBD is much less than those between 3G10 and RBD, the salt bridges formed between 3C11 and RBD may contribute to the high affinity comparable to that between 3G10 and RBD.

Both P.1 and B.1.351variants contain the K417T, E484K and N501Y mutations in RBD. E484K is a major mutation that is related to the immune escape of SARS-CoV-2. However, it is not involved in the interactions between 3G10 and RBD, but involved in the interactions between 3C11and RBD. This may explain the fact that 3G10 but not 3C11 neutralized Brazil and South Africa variants efficiently. Although K417 and N501 also contribute to the interactions between 3G10 and RBD, their mutations do not affect the binding of these two molecules considering these highly extensive atoms contacts. Since the end of November 2021, the SARS-CoV-2 Omicron variant (B.1.1.529), characterized by a high number of mutations in the S proteins, has spread worldwide and replaced other SARS-CoV-2 strains. The earliest sub-lineage of Omicron variant BA.1 (or B.1.1.529.1) harbors 13 mutations in RBD (G339D, S371L, S373P, S375F, K417N, N440K, G446S, S477N, T478K, E484A, Q493R, G496S, Q498R, N501Y, Y505H). Based on our crystal structures of prototype (PT) RBD in complex with 3G10 and 3C11, respectively, we can infer some clues of the reactivity of these two antibodies to Omicron BA.1 RBD. Among the Omicron BA.1-related mutations, G339D, S371L, S373P, S375F, N440K, G446S, and E484A are not located in the binding interface of 3G10 and RBD, so they do not affect 3G10 binding to RBD. On the other hand, K417N, T478K, Q493R, G496S, and Q498R may result in alteration in the hydrogen network, but their effect on 3G10 binding is unpredictable. However, N501Y may adversely affect 3G10 binding because the large hydrophobic side chain of phenylalanine would destroy the local interaction network. Y505H may also reduce 3G10 binding because the hydrogen bond between Y505 and 3G10 light chain S93 would lose. Therefore, the Omicron N501Y and Y505H can lead to the escape of Omicron from the neutralization of 3G10. As for 3C11, most BA.1 RBD mutations occur out of the binding interface so do not affect binding, but E484A would probably reduce binding because the salt bridge between E484 and 3C11 light chain R101 will lose. So, we presume that Omicron would escape both the antibodies.

High SHM rates and unique germlines of antibodies have been considered to play significant roles in influencing the neutralizing activity of antibodies. In this research, we found that most isolated neutralizing antibodies sequences have low SHM rates (<10%), implying that RBD-specific neutralizing antibodies may arise soon. In contrast to HIV broadly neutralizing antibodies targeting CD4 binding site, such as VRC01- or DRVIA7-like antibodies (39, 40), no separate islands of heavy or light chains of RBD-specific neutralizing antibodies were detected in 2D plots analysis, which is consistent with their short evolution period. Our findings are also in accord with a previous study on a convalescent of Zika infection. In that research, a neutralizing antibody—ZK2B10—was found to have a low-SHM level and no distinct islands were found in the identity-divergence two-dimensional (2D) plots (21). Regarding the germline utilization, germline alleles of the V_H_ of 1D7, 3G10 and 3C11 are IGHV1-69, IGHV3-66 and IGHV3-48, respectively. Some class I RBD-specific neutralizing antibodies were also derived from the IGHV3-66 germline allele (32). We also found that some potent neutralizing antibodies against SARS-CoV-2 WH-1 originate from pan-germline, which is consistent with previous researches (41).

There are some limitations to this research. First, the antibody-antigen complex structure of a broadly neutralizing antibody—1D7—was not successfully resolved for the crystal of 1D7/RBD complex was not harvested. As a result, we do not know which antibody class it belongs to or which amino acids are involved in the antigen-antibody interaction. Second, while we have isolated several neutralizing antibodies against SARS-CoV-2 using RBD, we have not investigated whether NTD-specific neutralizing antibodies exist in the antibody reservoir. Besides, further research to isolate elite neutralizing antibodies using an alternative method such as single-cell sequencing should be performed.

In conclusion, we have got three potent neutralizing antibodies against SARS-CoV-2 WH-1 and Delta authentic viruses. One of which, 1D7, demonstrated broadly neutralizing activity against WH-1, P.1, B.1.351, B.1.617.2 and B.1.1.529 authentic viruses. According to the antigen-antibody complex structure, 3G10 and 3C11 belong to RBD-1 and RBD-4 families, respectively. Antibody repertoire analysis also revealed that pan-germlines can produce RBD-specific neutralizing antibodies. These antibodies and antibody repertoires from these two donors will provide candidate for promising cocktail therapy as well as valuable information for vaccine design to treat or prevent SARS-CoV-2 infection.

## Data availability statement

The structures of 3G10-RBD and 3C11-RBD were deposited into the protein data bank with PDB IDs of 8HN6 and 8HN7, respectively. The original contributions presented in the study are included in the article/supplementary material. Further inquiries can be directed to the corresponding author/s.

## Ethics statement

The study was conducted in accordance with the Declaration of Helsinki, and approved by the Ethics Committee of the first affiliated hospital, Zhejiang University School of Medicine, China (approval number:2020-IIT-433). The patients/participants provided their written informed consent to participate in this study. Written informed consent was obtained from the subjects to publish this paper.

## Author contributions

Conceptualization, JQ, BZ, and YiS; methodology, ZW, DL, YC, YeS, CJ, SW, and MZ; software, YF, DL, and SW; validation, ZW, JQ, BZ, and YiS; formal analysis, ZW, DL, YC, YeS, and CJ; investigation, CH and JS; resources, JQ, BZ, and YiS; data curation, ZW, DL, YC, YeS, and CJ; writing—original draft preparation, ZW, DL, YC, and YeS; writing—review and editing, ZW, DL, JQ, and YeS; visualization, ZW, DL, YC, YeS, and YF; supervision, JQ, BZ, and YiS; project administration, JQ, BZ, and YiS; funding acquisition, ZW, JQ, and YiS. All authors contributed to the article and approved the submitted version.
