# Supplementary material for: Characterization of RBD-specific cross-neutralizing antibodies responses against SARS-CoV-2 variants from COVID-19 convalescents
Source: Front Immunol. 2023 May 10;14:1160283. doi: 10.3389/fimmu.2023.1160283 (PMC10207940; doi:10.3389/fimmu.2023.1160283)
Supplement: Supplementary file 1 [file DataSheet_1.pdf]

Table S1. Amino acids that are involved in the interaction between antibody 3C11 and RBD

3C11 to RBD			
Light chain		RBD	contacts(16)
FR2	52TYR	483VAL, 484GLU, 485GLY	5, 3, 2
LCDR2	53ASP	483VAL	1
FR3	56LYS	479PRO, 480CYS, 481ASN	1, 2, 1
LCDR3	94ARG	483VAL	1

3C11 to RBD			
Heavy chain		RBD	contacts(153)
FR1	1GLU	449TYR	1
HCDR1	26GLY	449TYR	22
	27PHE	449TYR	13
	28ALA	449TYR, 452LEU	4, 2
	31THR	490PHE	4
	32TYR	484GLU	2
FR3	74ASN	450ASN	1
	75ALA	346ARG, 450ASN	3, 3
	77ASN	450ASN	4
HCDR3	100LEU	483VAL,484GLU	3,9
	101ARG	472ILE,482GLY,483VAL,484GLU,490PHE	2,3,1,23,27
	102TYR	470THR,472ILE,482GLY,483VAL,490PHE	7,1,6,,1,7
	103SER	482GLY, 483VAL	1,2
	108ASP	484GLU	1

Table S2. Amino acids that are involved in the interaction between antibody 3G10 and RBD

3G10 to RBD			
Light chain		RBD	contacts(109)
FR1	2ILE	505TYR	4
LCDR1	27GLN	502GLY,503VAL	3, 1
	28GLY	501ASN, 502GLY, 500THR, 505TYR	11, 6, 4, 3
	29ILE	505TYR	4
	30SER	501ASN, 498GLN. 496GLY	6, 8, 6
	31THR	449TYR, 498GLN	2. 1
	32TYR	403ARG, 505TYR, 496GLY, 495TYR	2, 4, 9, 4
FR3	67SER	498GLN	4
	68GLY	498GLN, 500THR	1,1
LCDR3	90LEU	505TYR	4
	92ASN	417LYS, 403ARG, 453TYR	5, 5, 2
	93SER	403ARG, 505TYR	5, 4

3G10 to RBD			
Heavy chain		RBD	contacts(271)
FR1	2VAL	486PHE	5
HCDR1	26GLY	487ASN, 486PHE	4, 1
	27LEU	487ASN, 475ALA, 476GLY	5, 3, 2
	28THR	475ALA, 476GLY, 458LYS, 477SER	9,6,1,4
	30SER	458LYS	9
	31SER	458LYS,473TYR, 475ALA, 474GLN	11,9,3,2
	32ASN	475ALA	8
	33TYR	456PHE, 455LEU, 421TYR, 417LYS	4,9.3.3
HCDR2	52TYR	421TYR, 420ASP, 416GLY, 417LYS	7, 4, 7, 7
	53ALA	421TYR, 458LYS, 457ARG, 473ARG	4, 8, 2, 1
	54GLY	421TYR,458LYS,460ASN,457ARG,459SER	5,4,10,1,2
	56SER	420ASP, 415THR, 416GLY, 460ASN	6, 6, 1, 1
FR3	58PHE	416GLY, 415THR	4, 7
HCDR3	97ARG	487ASN, 489TYR, 486PHE, 475ALA	5, 5, 4, 1
	99LEU	456PHE, 455LEU, 489TYR	9, 1,10
	100SER	455LEU, 417LYS	2,1
	101TYR	455LEU,493GLN, 453TYR, 494SER	2,26,1,1
	102TYR	456PHE, 493GLN, 455LEU	3, 5, 1
	105ASP	486PHE, 489TYR	3,1
	106VAL	486PHE	2

Figure S1. Characterization of RBD-specific antibody response and neutralizing activity in convalescent plasma (A) RBD-specific responses of convalescent plasma. The binding capacity is indicated as optical density (OD) measured at 450 nm in the Y-axis and plasma reciprocal dilution in the X-axis. Plasma from healthy people was set as negative control. The results are shown as averages with standard deviations of triplicate experiments. (B) The neutralizing activity of convalescent plasma against WT pseudovirus.

Figure S2. Neutralizing activity (IC50, μg/mL) of isolated antibodies against WH-1 virus （A） Antibodies of donor CZ; (B) Antibodies of donor WJQ.

Figure S3. Alignment of heavy chain or light chain of neutralizing antibodies with their germlines. A. V_H and Vκ of 1D7, B. V_H and Vκ of 3G10, C. V_H and Vκ of 3C11.

Figure S4. Antibodyomics analysis of the donor CZ by unbiased deep-sequencing. (A) Germline distributions of Heavy chains, kappa chains and lambda chains. The x-axis indicates the distribution of germlines, whereas the y-axis indicates the proportion of acquired sequences. (B) Percentages of sequences with different somatic hypermutation rates in the whole

repertoire. The x-axis represents the SHM rates and the y-axis indicates the sequence proportion. (C) Analysis of CDR3 loop lengths of different germlines. The x-axis represents the CDR3 lengths of the heavy chains, κ chains and λ chains, respectively. The y-axis indicates the proportion of sequences with diverse CDR3 lengths.

Figure S5. Antibody repertoire of the donor WJQ by deep-sequencing. (A) Germline distributions of Heavy chains, kappa chains and lambda chains. (B) Percentages of sequences with different somatic hypermutation rates in the whole repertoire. The x-axis represents the SHM rates and the y-axis indicates the sequence proportion. (C) CDR3 loop lengths of each germline.

Figure S6. Chord diagram representation of V and J frequency and their associations in antibody repertoires. The size of the inner segments corresponds to the number of sequences of each gene, and the diagrams are color-coded to indicate the pairing network of the VH and VJ. (A) Donor CZ. (B) Donor WJQ.

Figure S7. Neutralizing activity of isolated antibodies against BA.5, BF.7 and XBB, respectively.

A

Fig S1

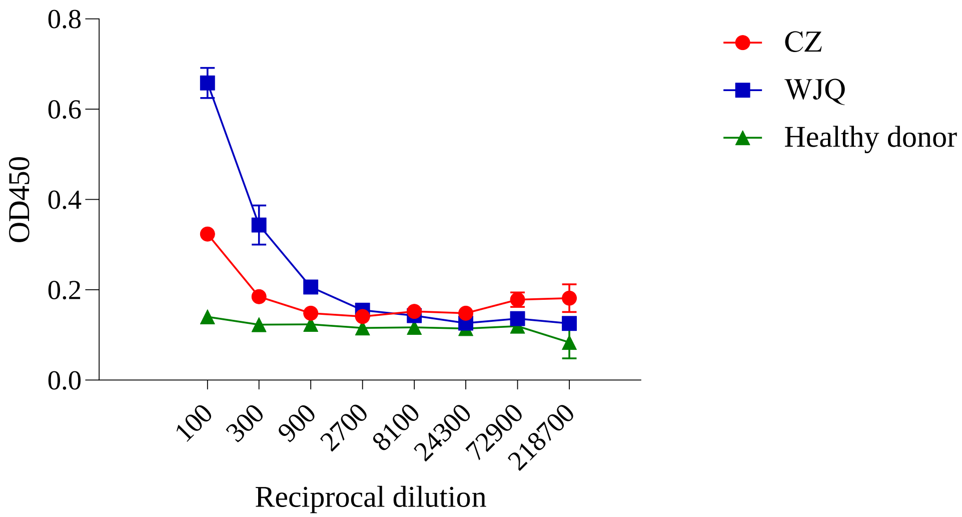

0.8
0.6
0.4
0.2
0.0
OD450
100
300
900
2700
8100
24300
72900
218700
Reciprocal dilution
CZ
WJQ
Healthy donor

B

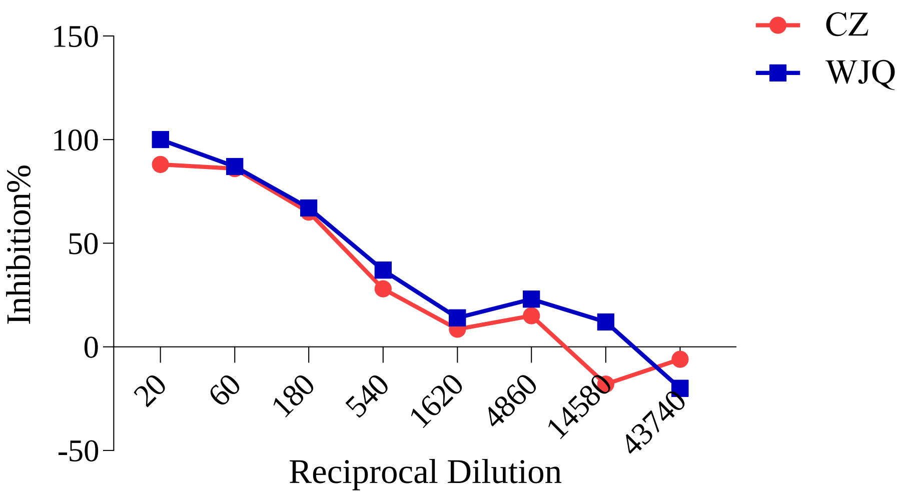

150
100
50
0
-50
Inhibition%
20
60
180
540
1620
4860
14580
43740
Reciprocal Dilution
CZ
WJQ

Fig S2

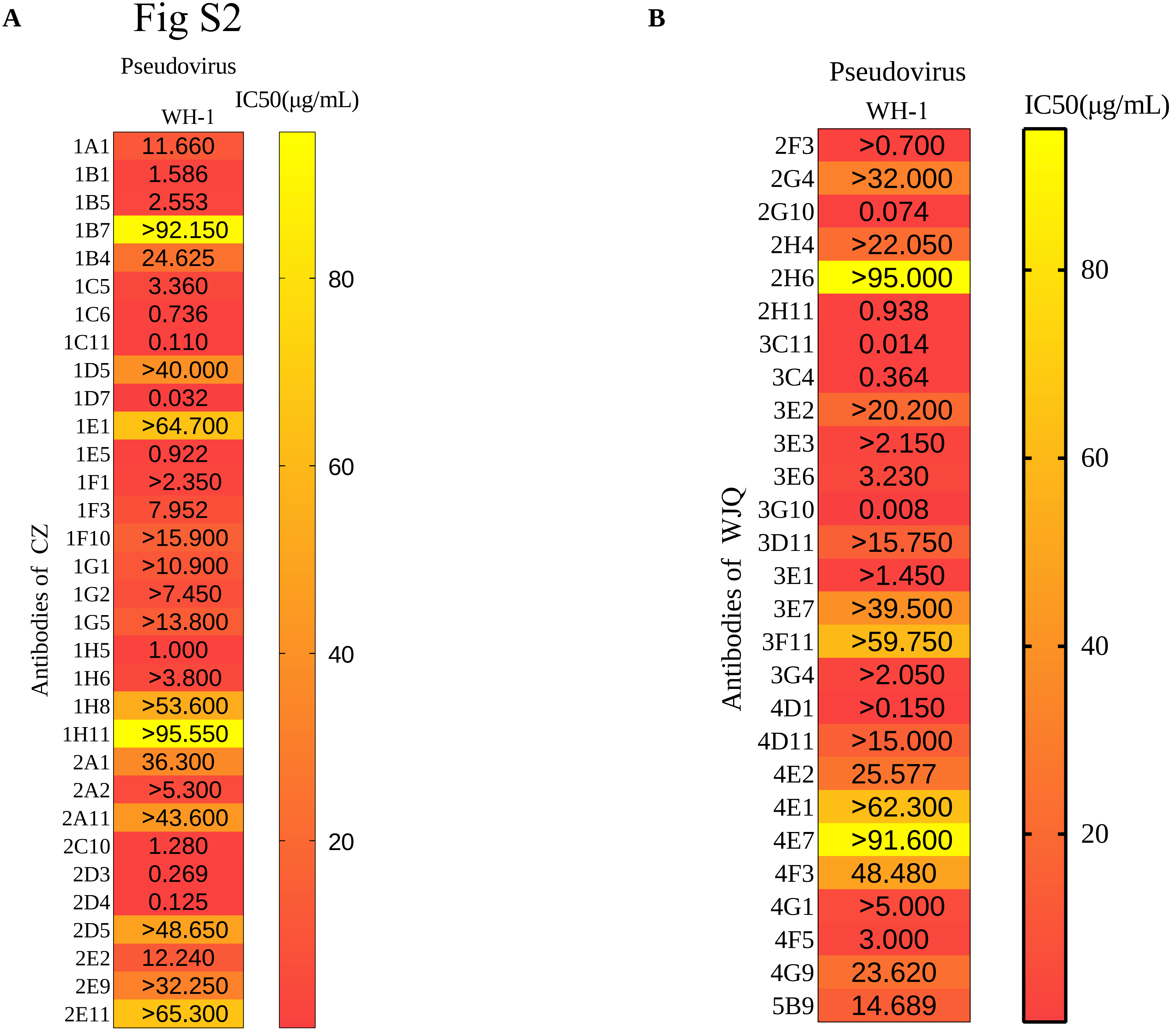
A
Pseudovirus
WH-1
IC50(μg/mL)
Antibodies of CZ
1A1 11.660
1B1 1.586
1B5 2.553
1B7 >92.150
1B4 24.625
1C5 3.360
1C6 0.736
1C11 0.110
1D5 >40.000
1D7 0.032
1E1 >64.700
1E5 0.922
1F1 >2.350
1F3 7.952
1F10 >15.900
1G1 >10.900
1G2 >7.450
1G5 >13.800
1H5 1.000
1H6 >3.800
1H8 >53.600
1H11 >95.550
2A1 36.300
2A2 >5.300
2A11 >43.600
2C10 1.280
2D3 0.269
2D4 0.125
2D5 >48.650
2E2 12.240
2E9 >32.250
2E11 >65.300
80
60
40
20
B
Pseudovirus
WH-1
IC50(μg/mL)
Antibodies of WJQ
2F3 >0.700
2G4 >32.000
2G10 0.074
2H4 >22.050
2H6 >95.000
2H11 0.938
3C11 0.014
3C4 0.364
3E2 >20.200
3E3 >2.150
3E6 3.230
3G10 0.008
3D11 >15.750
3E1 >1.450
3E7 >39.500
3F11 >59.750
3G4 >2.050
4D1 >0.150
4D11 >15.000
4E2 25.577
4E1 >62.300
4E7 >91.600
4F3 48.480
4G1 >5.000
4F5 3.000
4G9 23.620
5B9 14.689
80
60
40
20

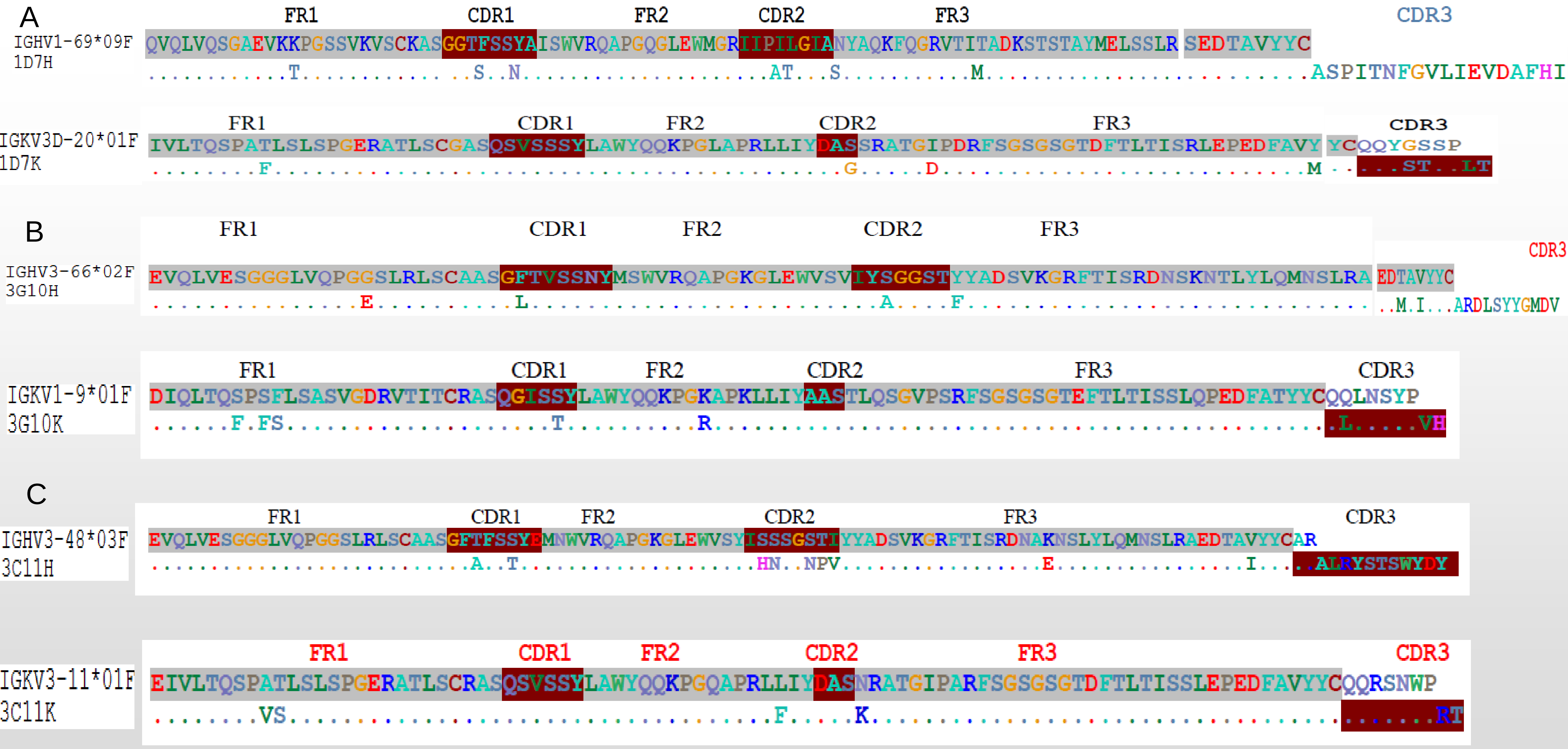
A
FR1 CDR1 FR2 CDR2 FR3 CDR3
IGHV1-69*09F
1D7H
QVQLVQSGAEVKKPGSSVKVSCKASGGTFSSYAISWVRQAPGQGLEWMGRIIPILGIANYAQKFQGRVTITADKSTSTAYMELSSLR SEDTAVYYC
...........T.......... ..S..N.........................AT...S..........M..........................ASPITNFGVLIEVDAFHI
FR1 CDR1 FR2 CDR2 FR3 CDR3
IGKV3D-20*01F
1D7K
IVLTQSPATLSLSPGERATLSCGASQSVSSSYLAWYQQKPGLAPRLLIYDASSRATGIPDRFSGSGSGTDFTLTISRLEPEDFAVYYCQQYGSSP
........F...G....D..........................M - - ...ST..LT
B
FR1 CDR1 FR2 CDR2 FR3 CDR3
IGHV3-66*02F
3G10H
EVQLVESGGGLVQPGGSLRLSCAASGFTVSSNYMSWVRQAPGKGLEWVSVIYSGGSTYYADSVKGRFTISRDNSKNTLYLQMNSLRA EDTAVYYC
...............E..........L.........................A....F.............................. ..M.I...ARDLSYYGMDV
FR1 CDR1 FR2 CDR2 FR3 CDR3
IGKV1-9*01F
3G10K
DIQLTQSPSFLSASVGDRVTITCRASQGISSYLAWYQQKPGKAPKLLIYAASTLQSGVPSRFSGSGSGTEFTLTISSLQPEDFATYYCQQLNSYP
......F.FS...................T..........R..L.....VH
C
FR1 CDR1 FR2 CDR2 FR3 CDR3
IGHV3-48*03F
3C11H
EVQLVESGGGLVQPGGSLRLSCAASGFTFSSYEMNWVRQAPGKGLEWVSYISSSGSTIYYADSVKGRFTISRDNAKNSLYLQMNSLRAEDTAVYYCAR
...........................A..T..................HN..NPV................E................I.....ALRYSTSWYDY
FR1 CDR1 FR2 CDR2 FR3 CDR3
IGKV3-11*01F
3C11K
EIVLTQSPATLSLSPGERATLSCRASQSVSSYLAWYQQKPGQAPRLLIYDASNRATGIPARFSGSGSGTDFTLTISSLEPEDFAVYYCQQRSNWP
........VS..............................F.....K...RT

Fig S3

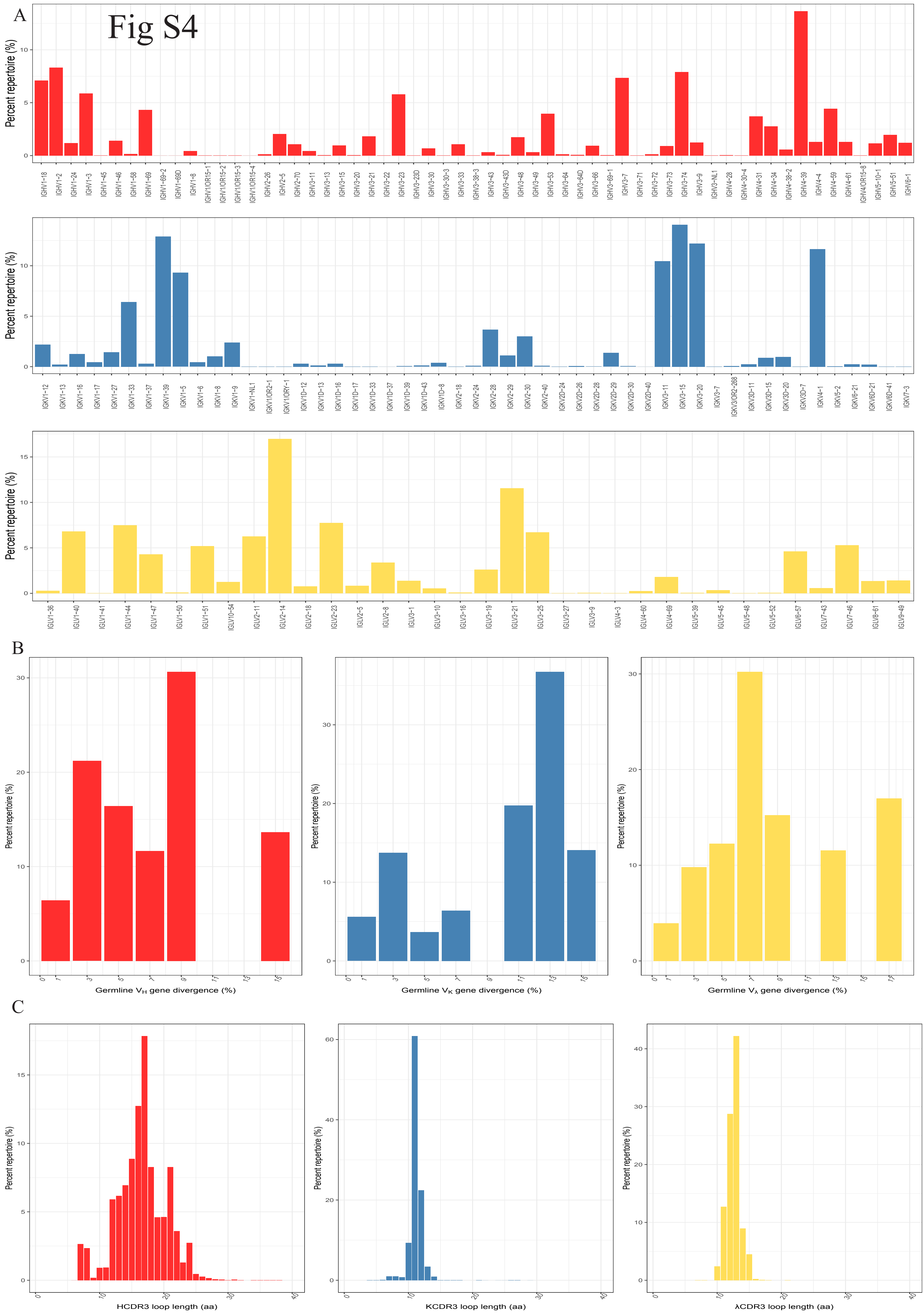
Fig S4
A
Percent repertoire (%)
IGHV1-18 IGHV1-2 IGHV1-24 IGHV1-3 IGHV1-45 IGHV1-46 IGHV1-58 IGHV1-69 IGHV1-69-2 IGHV1-69D IGHV1-8 IGHV1/OR15-1 IGHV1/OR15-2 IGHV1/OR15-3 IGHV1/OR15-4 IGHV2-26 IGHV2-5 IGHV2-70 IGHV3-11 IGHV3-13 IGHV3-15 IGHV3-20 IGHV3-21 IGHV3-22 IGHV3-23 IGHV3-23D IGHV3-30 IGHV3-30-3 IGHV3-33 IGHV3-38-3 IGHV3-43 IGHV3-43D IGHV3-48 IGHV3-49 IGHV3-53 IGHV3-64 IGHV3-64D IGHV3-66 IGHV3-69-1 IGHV3-7 IGHV3-71 IGHV3-72 IGHV3-73 IGHV3-74 IGHV3-9 IGHV3-NL1 IGHV4-28 IGHV4-30-4 IGHV4-31 IGHV4-34 IGHV4-38-2 IGHV4-39 IGHV4-4 IGHV4-59 IGHV4-61 IGHV4/OR15-8 IGHV5-10-1 IGHV5-51 IGHV6-1
Percent repertoire (%)
IGKV1-12 IGKV1-13 IGKV1-16 IGKV1-17 IGKV1-27 IGKV1-33 IGKV1-37 IGKV1-39 IGKV1-5 IGKV1-6 IGKV1-8 IGKV1-9 IGKV1-NL1 IGKV1/OR2-1 IGKV1/ORY-1 IGKV1D-12 IGKV1D-13 IGKV1D-16 IGKV1D-17 IGKV1D-33 IGKV1D-37 IGKV1D-39 IGKV1D-43 IGKV1D-8 IGKV2-18 IGKV2-24 IGKV2-28 IGKV2-29 IGKV2-30 IGKV2-40 IGKV2D-24 IGKV2D-26 IGKV2D-28 IGKV2D-29 IGKV2D-30 IGKV2D-40 IGKV3-11 IGKV3-15 IGKV3-20 IGKV3-7 IGKV3/OR2-268 IGKV3D-11 IGKV3D-15 IGKV3D-20 IGKV3D-7 IGKV4-1 IGKV5-2 IGKV6-21 IGKV6D-21 IGKV6D-41 IGKV7-3
Percent repertoire (%)
IGLV1-36 IGLV1-40 IGLV1-41 IGLV1-44 IGLV1-47 IGLV1-50 IGLV1-51 IGLV10-54 IGLV2-11 IGLV2-14 IGLV2-18 IGLV2-23 IGLV2-5 IGLV2-8 IGLV3-1 IGLV3-10 IGLV3-16 IGLV3-19 IGLV3-21 IGLV3-25 IGLV3-27 IGLV3-9 IGLV4-3 IGLV4-60 IGLV4-69 IGLV5-39 IGLV5-45 IGLV5-48 IGLV5-52 IGLV6-57 IGLV7-43 IGLV7-46 IGLV8-61 IGLV9-49
B
Percent repertoire (%)
Germline VH gene divergence (%)
Germline VK gene divergence (%)
Germline Vλ gene divergence (%)
C
HCDR3 loop length (aa)
KCDR3 loop length (aa)
λCDR3 loop length (aa)

Fig S5

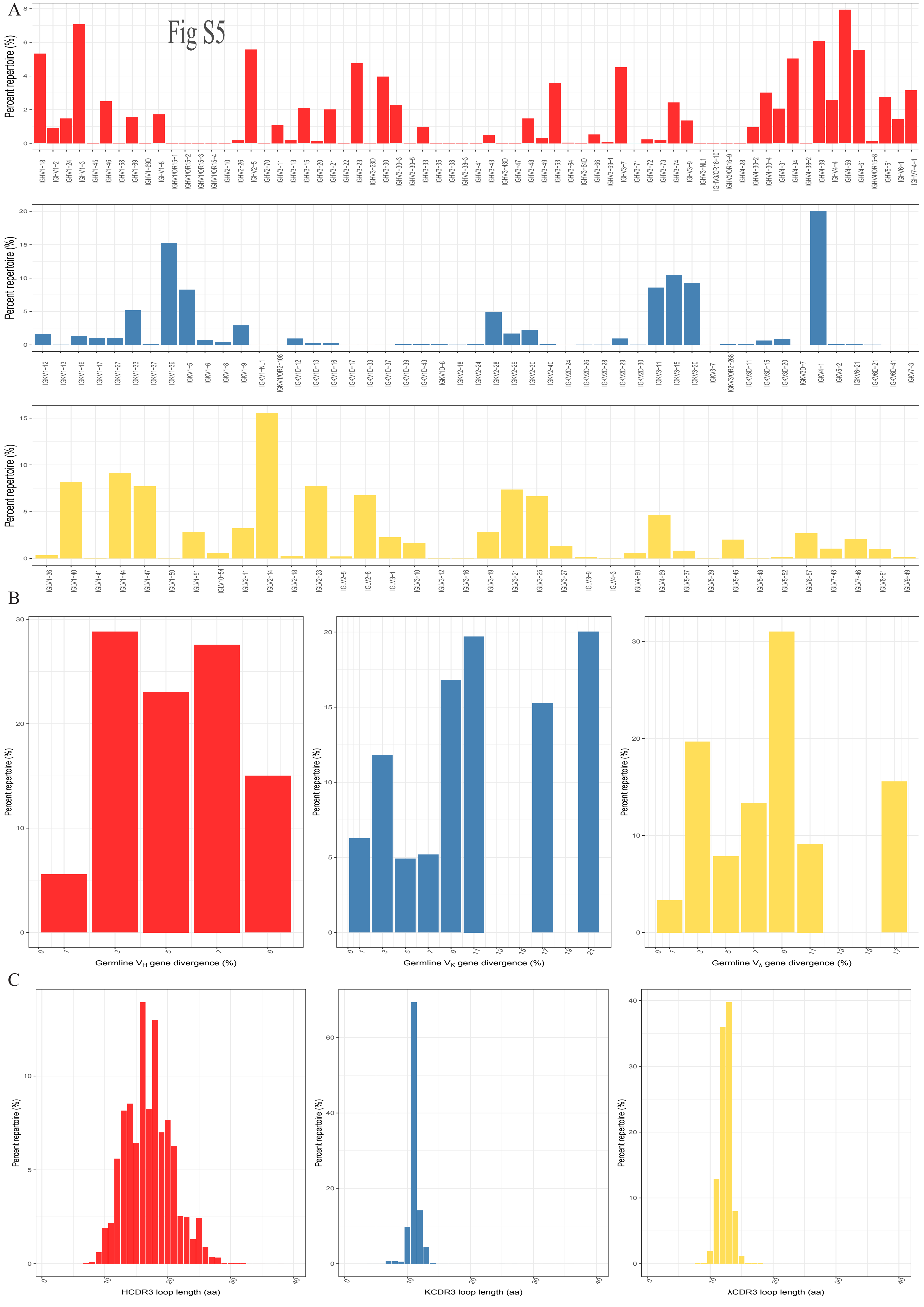

A
Percent repertoire (%)
Percent repertoire (%)
Percent repertoire (%)
B
Percent repertoire (%)
Germline V_H gene divergence (%)
Percent repertoire (%)
Germline V_K gene divergence (%)
Percent repertoire (%)
Germline V_λ gene divergence (%)
C
Percent repertoire (%)
HCDR3 loop length (aa)
Percent repertoire (%)
KCDR3 loop length (aa)
Percent repertoire (%)
λCDR3 loop length (aa)

Figure S6

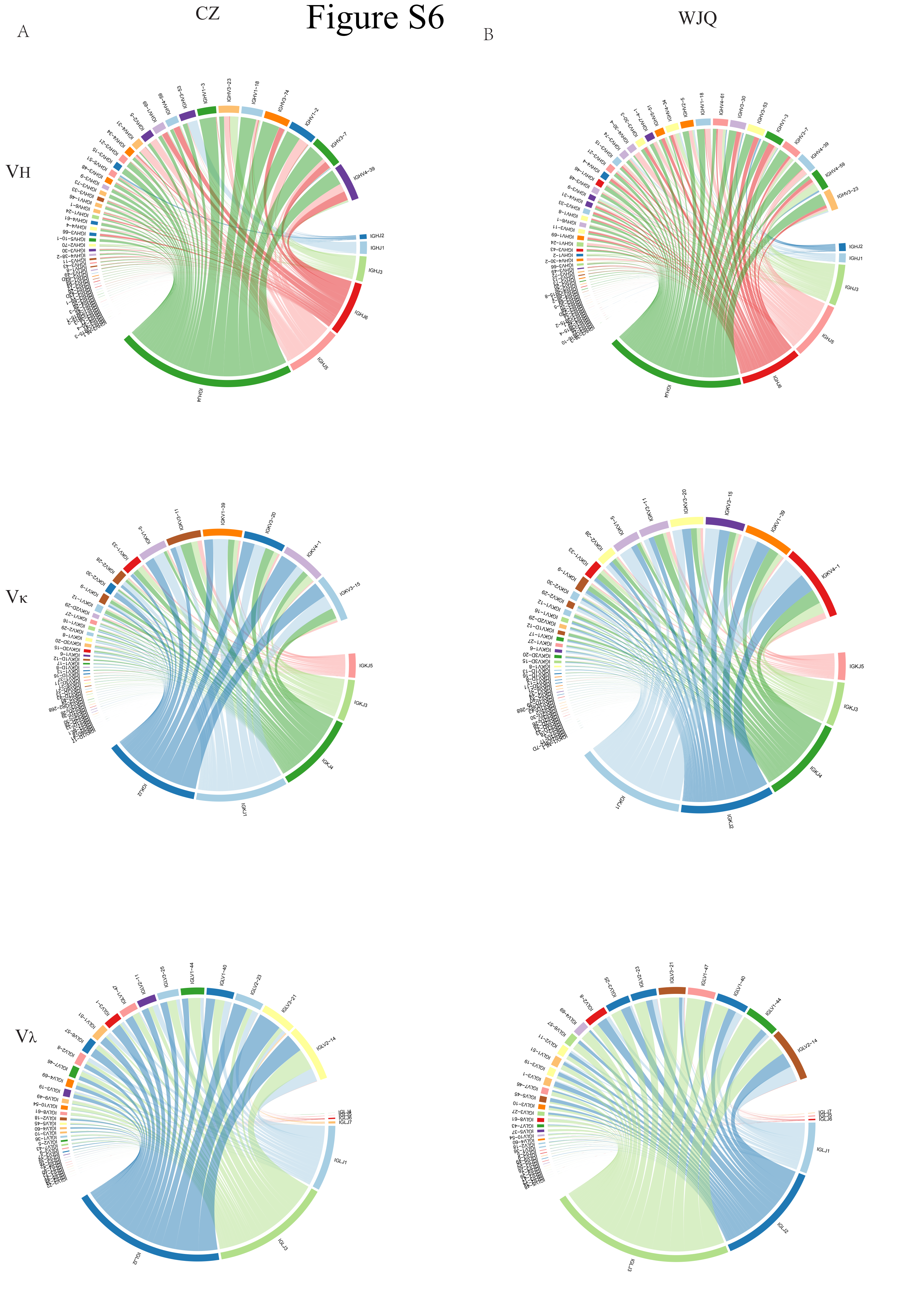

Figure S7

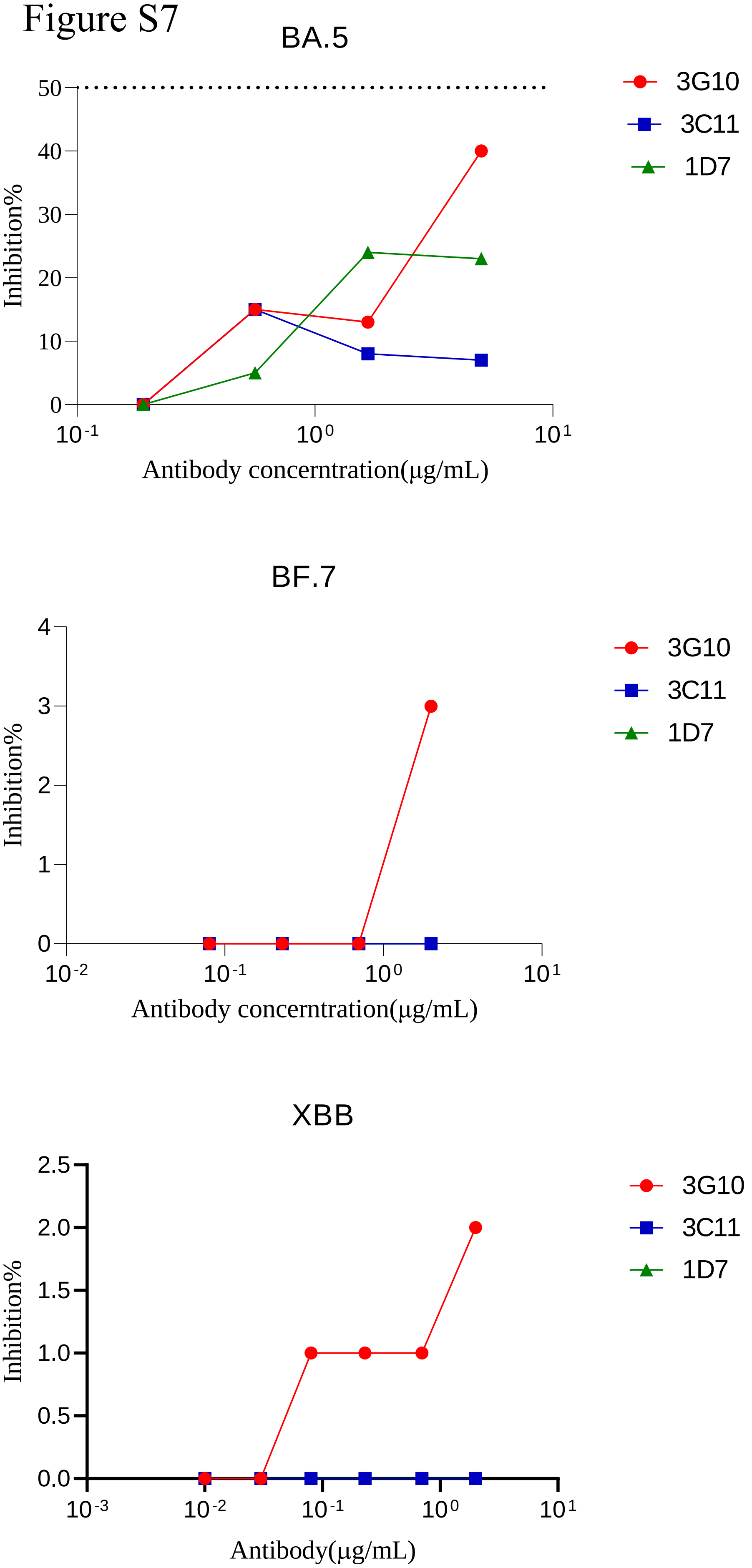

BA.5
Inhibition%
Antibody concenrtration(μg/mL)
3G10
3C11
1D7
BF.7
Inhibition%
Antibody concenrtration(μg/mL)
3G10
3C11
1D7
XBB
Inhibition%
Antibody(μg/mL)
3G10
3C11
1D7
